# Microcirculatory consequences of limb ischemia/reperfusion in ovariectomized rats treated with zoledronic acid

**DOI:** 10.1186/s13018-019-1117-x

**Published:** 2019-04-04

**Authors:** Levente Pócs, Ágnes Janovszky, Imre Ocsovszki, József Kaszaki, József Piffkó, Andrea Szabó

**Affiliations:** 1 Department of Traumatology and Hand Surgery, Bács-Kiskun County Teaching Hospital, Nyíri u. 38, Kecskemét, H-6000 Hungary; 2 Department of Oral and Maxillofacial Surgery, University of Szeged, Kálvária sgt. 57, Szeged, H-6725 Hungary; 3 Department of Biochemistry, University of Szeged, Dóm tér 9, Szeged, H-6720 Hungary; 4 Institute of Surgical Research, University of Szeged, Pulz u. 1, Szeged, H-6724 Hungary

**Keywords:** Bisphosphonate, Periosteum, Inflammation, Intravital microscopy, Leukocytes

## Abstract

**Background:**

Nitrogen-containing bisphosphonates (BIS) are potent therapeutics in osteoporosis, but their use may result in osteonecrotic side-effects in the maxillofacial region. Periosteal microcirculatory reactions may contribute to the development of bone-healing complications, particularly in osteoporotic bones, where ischemia–reperfusion (IR) events often develop during orthopaedic/trauma interventions. The effect of BIS on the inflammatory reactions of appendicular long bones has not yet been evaluated; thus, we aimed to examine the influence of chronic zoledronate (ZOL) administration on the periosteal microcirculatory consequences of hindlimb IR in osteopenic rats.

**Materials and methods:**

Twelve-week-old female Sprague–Dawley rats were ovariectomized (OVX) or sham-operated, and ZOL (80 μg/kg iv, weekly) or a vehicle was administered for 8 weeks, 4 weeks after the operation. At the end of the pre-treatment protocols, 60-min limb ischemia was induced, followed by 180-min reperfusion. Leukocyte-endothelial interactions were quantitated in tibial periosteal postcapillary venules by intravital fluorescence videomicroscopy. CD11b expression of circulating polymorphonuclear leukocytes (PMN, flow cytometry) and plasma TNF-alpha levels (ELISA) were also determined. Two-way RM ANOVA followed by the Holm–Sidak and Dunn tests was used to assess differences within and between groups, respectively.

**Results:**

Limb IR induced significant increases in PMN rolling and firm adhesion in sham-operated and OVX rats, which were exacerbated temporarily in the first 60 min of reperfusion by a ZOL treatment regimen. Postischemic TNF-alpha values showed a similar level of postischemic elevations in all groups, whereas CD11b expression only increased in rats not treated with ZOL.

**Conclusions:**

The present data do not show substantial postischemic periosteal microcirculatory complications after chronic ZOL treatment either in sham-operated or OVX rats. The unaltered extent of limb IR-induced local periosteal microcirculatory reactions in the presence of reduced CD11b adhesion molecule expression on circulating PMNs, however, may be attributable to local endothelial injury/activation caused by ZOL.

## Introduction

Osteoporosis affects more than 75 million people worldwide [1], with every other woman and every fifth man over 50 years suffering an osteoporotic fracture of the extremities during her or his remaining lifetime [2]. Ischemia–reperfusion (IR) often takes place in the affected tissue, e.g. due to use of a tourniquet or other temporary occlusive devices and techniques during trauma surgeries. In these cases, IR can lead to unwanted complications through the development of an antigen-independent inflammatory process, which involves the activation and adhesion of polymorphonuclear (PMN) leukocytes in the periosteal endothelium [3] and the upregulation of several danger-associated molecular pattern-related pathways in the locally affected and contralateral limbs as well [4]. Proinflammatory cytokines (e.g. TNF-alpha, IL-1 and IL-6) typically reach peak values after fracture operations [5], and it is suggested that these proinflammatory reactions may critically influence the process of bone regeneration.

Bisphosphonates (BISs) are potent therapeutic agents that ameliorate the osteoporosis-induced decrease of bone mineral density [2, 6]. Further, it has been shown that the risk of osteoporotic fractures can be reduced, in particular with the use of zoledronic acid (ZOL) [2, 7]. Nevertheless, BIS-induced unwanted, necrotic reactions may also be present in the skeletal system. Specifically, chronic BIS treatment can effectively enhance the incorporation of bone implants in appendicular bones [8, 9], but the likelihood of osteonecrotic complications also increases in parallel at the jaw bones [2, 10, 11]. The incidence of necrosis is especially high in the mandible after oral surgical interventions, leading to a condition termed medication-related osteonecrosis of the jaws. An exact pathogenesis of this complication is still unknown, but it seems to affect the appendicular and axial bones differently [12, 13]. Previously, we have also shown that BISs can induce significant inflammatory reactions in the mandibular periosteum after tooth extractions, while the microcirculation in the tibial region remained unaffected [14].

The aim of the present study was to examine the effects of chronic BIS treatment on the postischemic periosteal microcirculatory changes of the lower extremities. To our knowledge, the possible modulator role of BIS on the inflammatory reactions of the appendicular bones disposed to IR injuries has not yet been evaluated. Based on the relevant literature data and our previous results, our null hypothesis was that ZOL treatment does not influence the periosteal microcirculatory reactions of transient limb IR. We tested this hypothesis in a clinically relevant model of osteoporosis where anaesthetized rats were challenged with standardised limb IR in the presence or absence of chronic ZOL treatment.

## Materials and methods

All studies were carried out on Sprague–Dawley rats housed in an environmentally controlled room with a 12-h light–dark cycle. The animals were kept on commercial rat chow (Charles River, Wilmington, MA, USA) and tap water ad libitum.

The project was approved by the National Scientific Ethics Committee on Animal Experimentation (National Competent Authority) under licence number V./144/2013. The study was performed in compliance with EU Directive 2010/63/EU on the protection of animals used for experimental and other scientific purposes and the National Institutes of Health guidelines on the use of experimental animals. Animal welfare-related assessments and interventions were carried out prior to and during the experiments.

### Experimental protocol

#### Ovariectomy

Ovariectomy (OVX) is a well-established animal model of osteoporosis sharing many similarities with the human condition including increased rate of bone turnover, relatively rapid bone loss and most importantly, similar skeletal responses to treatments used in humans (e.g. oestrogen, calcitonin and BISs) [15, 16]. In our study, 12-week-old female rats (weighing 180 to 200 g) were randomly allocated to ovariectomized (*N* = 32) or sham-operated (*N* = 30) groups under anaesthesia administered intraperitoneally by a combination of ketamine and xylazine (25 mg kg^−1^ and 75 mg kg^−1^, respectively). As conditions were sterile, a median laparotomy was performed and the connection of the Fallopian tubes cut between haemostats. The ovaries were then removed and the stumps ligated with a 3-0 non-absorbable thread (Ethibond Excel®, Ethicon, Somerville, NJ, USA). Thereafter, the abdomen was filled with warm sterile physiological saline, and the abdominal wall was closed with a 4-0 absorbable suture and a 4-0 non-absorbable suture (Vicryl® and Prolene®, Ethicon, Somerville, NJ, USA) in two layers. Sham-operated animals underwent identical procedures, except of course that the Fallopian tubes and ovaries were not touched.

#### Experimental protocol, experimental groups

**Fig. 1:**
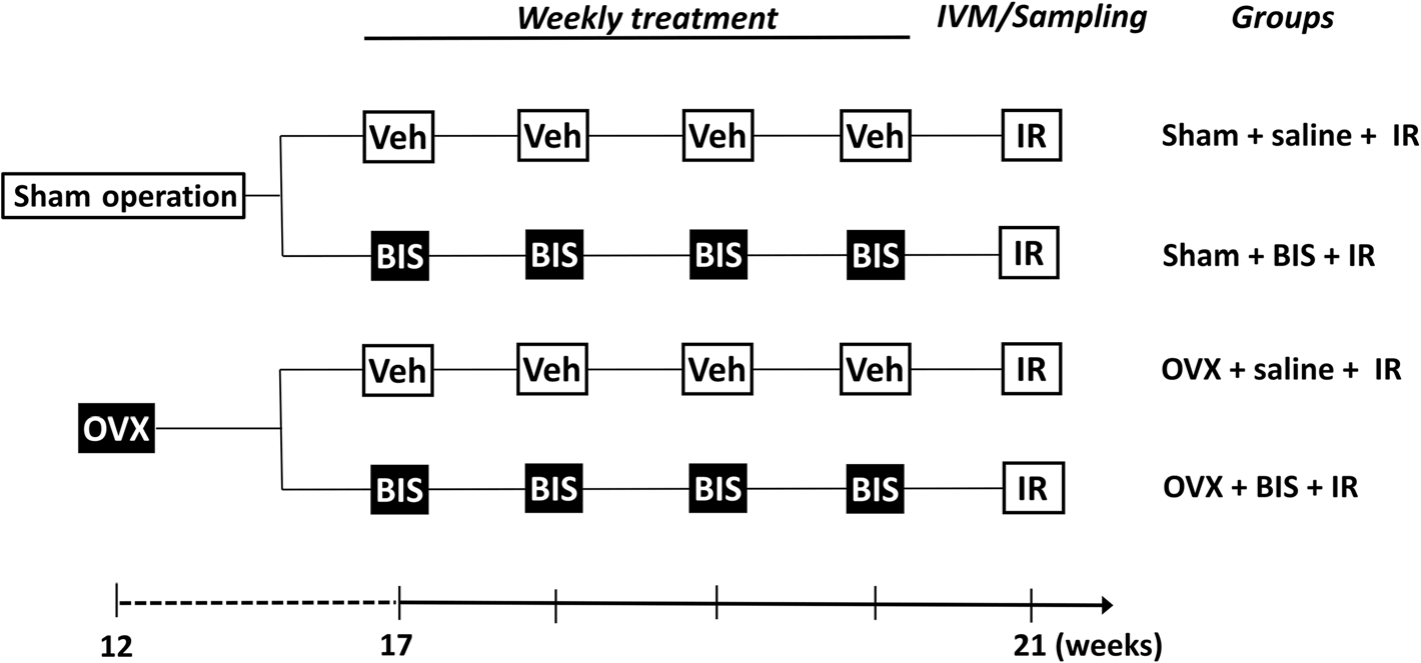
Time sequence of interventions and treatments. Rats were sham-operated or ovariectomized (OVX) at the age of 12 weeks. Five weeks later (on week 17), chronic bisphosphonate (BIS, once a week in a dose of 80 μg kg^−1^ in the tail vein) or saline vehicle (Veh) treatment was initiated. At the end of the protocol (week 21), 60 min of limb ischemia followed was induced by 180 min reperfusion (IR), and periosteal microcirculatory measurements by fluorescence intravital microscopy (IVM; series 1, *n* = 7–9) were conducted as well as sampling for leukocyte adhesion molecule CD11b expression and serum TNF-alpha measurements (series 2, *n* = 7 each)

Five weeks after OVX (i.e. at 17 weeks of age) (see Fig. 1), a chronic zoledronate treatment was initiated in 16 animals (OVX + BIS group) with 14 of the sham-operated animals serving as negative controls (Sham + BIS group). ZOL (80 μg kg^−1^ Zometa®, Novartis Europharm, Budapest, Hungary) was administered once a week intravenously into the tail vein under light aether anaesthesia. The remaining OVX and sham-operated animals received physiological saline in the same volume (OVX + vehicle and sham + vehicle groups, *n* = 16 each). These weekly injections were continued for 4 weeks. At the end of the experimental protocol (in week 21), all of the animals were subjected to a 60-min complete hindlimb ischemia followed by a 180-min reperfusion period. Limb ischemia was induced by applying a tourniquet around the thigh and placing a miniclip on the femoral artery. The experiments were performed in two experimental series. In series 1, the periosteal microcirculation was examined using fluorescence intravital microscopy (IVM) at baseline and every 60 min during the 180-min reperfusion period (*n* = 7–9 per group). In the second experimental series, blood samples from the carotid artery were taken at baseline and during the reperfusion period to detect changes in the plasma concentrations of TNF-alpha and in the expression of the adhesion molecule CD11b (*n* = 7 in each group). It was necessary to separate the two series to avoid any interference between the fluorescent dyes used for IVM and acquisition techniques used with flow cytometry.

#### Surgical procedure for intravital microscopic examination of the periosteal microcirculation

The animals were anaesthetized intraperitoneally with an initial dose of sodium pentobarbital (45 mg kg^−1^). After tracheal intubation, one of the jugular veins was also cannulated to administer fluid and drugs (supplementary dose of sodium pentobarbital, 5 mg kg^−1^). During the surgical procedures and investigation, the rats were placed in a supine position on a heating pad to maintain body temperature at 36–37 °C.

Under an operating microscope at × 4 magnification, the anteromedial surfaces of the tibial periosteum and the femoral artery on the same hindlimb were exposed with an atraumatic microsurgical technique [3]. The limbs were positioned horizontally on a special stage to expose periosteal vessels suitable for intravital fluorescence microscopy (IVM) in different phases of the experiment. At the end of the experiment, the animals were sacrificed with an overdose of sodium pentobarbital.

#### Intravital video microscopy

Leukocyte−endothelial cell interactions are decisive events among the complications of IR injury. After an initial low affinity interaction (i.e. rolling), a higher affinity binding (firm adhesion) takes place between the PMN and the endothelial surface. These dynamic cellular reactions can be detected in real time using IVM. In our model, the tibial periosteum was superfused with 37 °C saline, and the microcirculation was visualised by IVM (Zeiss Axiotech Vario 100HD microscope; Carl Zeiss GmbH, Jena, Germany, 100-W HBO mercury lamp, Acroplan 20x water immersion objective). Fluorescein isothiocyanate-labelled erythrocytes (0.2 ml intravenously; Sigma, St. Louis, MO, USA) were used to stain red blood cells, and rhodamine-6G (0.2%, 0.1 ml intravenously; Sigma, St. Louis, MO, USA) was used to label leukocytes [3]. The images from three to four fields of the tibial periosteum were recorded with a charge-coupled device video camera (Teli CS8320Bi, Toshiba Teli Corporation, Osaka, Japan), which is attached to an S-VHS video recorder (Panasonic AG-MD 830; Matsushita Electric Industrial Co., Tokyo, Japan) and a personal computer.

#### Video analysis

Quantitative evaluation of the microcirculatory parameters was performed offline by frame-to-frame analysis of the videotaped images (IVM; Pictron, Budapest, Hungary). Leukocyte–endothelial cell interactions were analysed in at least four postcapillary venules per rat. Rolling leukocytes were defined as cells moving with a velocity of less than 40% of that of the erythrocytes in the centreline of the microvessel passing through the observed vessel segment within 30 s and are given as the number of cells per millimetres per second. Adherent leukocytes were defined as cells that did not move or detach from the endothelial lining within an observation period of 30 s and are given as the number of cells per square millimetre of endothelial surface, calculated from the diameter and length of the vessel segment [17].

### Biochemical measurements

#### Immune labelling and flow cytometric analysis of CD11b expression of neutrophil leukocytes

Leukocyte–endothelial cell interactions are dependent upon simultaneously increased expression of adhesion molecules on the surfaces of PMNs and those of the activated endothelia. The firm adhesion step is mediated by PMN-derived ß2 integrins (i.e. CD11a, b, c/CD18) which bind to various integrin receptors (intercellular adhesion molecules-1 and intercellular adhesion molecules-2 and vascular adhesion molecule-1) of the endothelium [18]. An increased expression of CD11b on PMNs in the systemic circulation can be detected after limb IR [3]. In the present study, these changes were determined from the peripheral blood by flow cytometry in duplicate samples. One hundred microlitres of whole blood was incubated with 20 μl of FITC-conjugated mouse anti-rat CD11b monoclonal antibody (BD Pharmingen, San Jose, CA, USA) for 20 min. Negative controls were obtained by omitting the antibody. The cells were then washed twice in Hanks’ buffer and centrifuged for 5 min at 13,500 rpm, and the pellet was resuspended. The erythrocytes were lysed with a lysing kit (Biodesign, Saco, ME, USA), after which the cells were washed twice again (5 min, 6000 rpm) and resuspended in 750 μl of Hanks’ buffer. Computer-assisted FACStar Plus Becton Dickinson equipment was used for cytometry; the granulocytes were gated on the basis of their characteristic forward and side-scatter features. Generally, 10,000 events per sample were collected and recorded; the percentage of labelled (activated) granulocytes (relative to the overall marker-bearing cells) and the mean fluorescence intensity (average marker density) were calculated.

#### Detection of plasma level of TNF-alpha levels

The rest of the blood samples (0.5 ml) were centrifuged for 5 min at 4 °C at 13,500 rpm and then stored at 70 °C until assay. Plasma TNF-apha concentrations were determined in duplicate with a commercially available ELISA kit (Quantikine ultrasensitive ELISA kit for rat TNF-alpha; R&D Systems Inc., Minneapolis, MN, USA). The minimum detectable level was less than 5 pgml-1, and the inter-assay and intra-assay coefficients of variation were less than 10%.

### Statistical analysis

Data analysis was performed with a statistical software package (SigmaStat for Windows, Jandel Corporation, San Rafael, CA, USA) using nonparametric methods. Two-way RM ANOVA followed by the Holm–Sidak and Dunn tests was used to assess differences within and between groups, respectively. Data are presented as mean value and SEM in all figures. *P* values < 0.05 were considered significant.

## Results

**Fig. 2:**
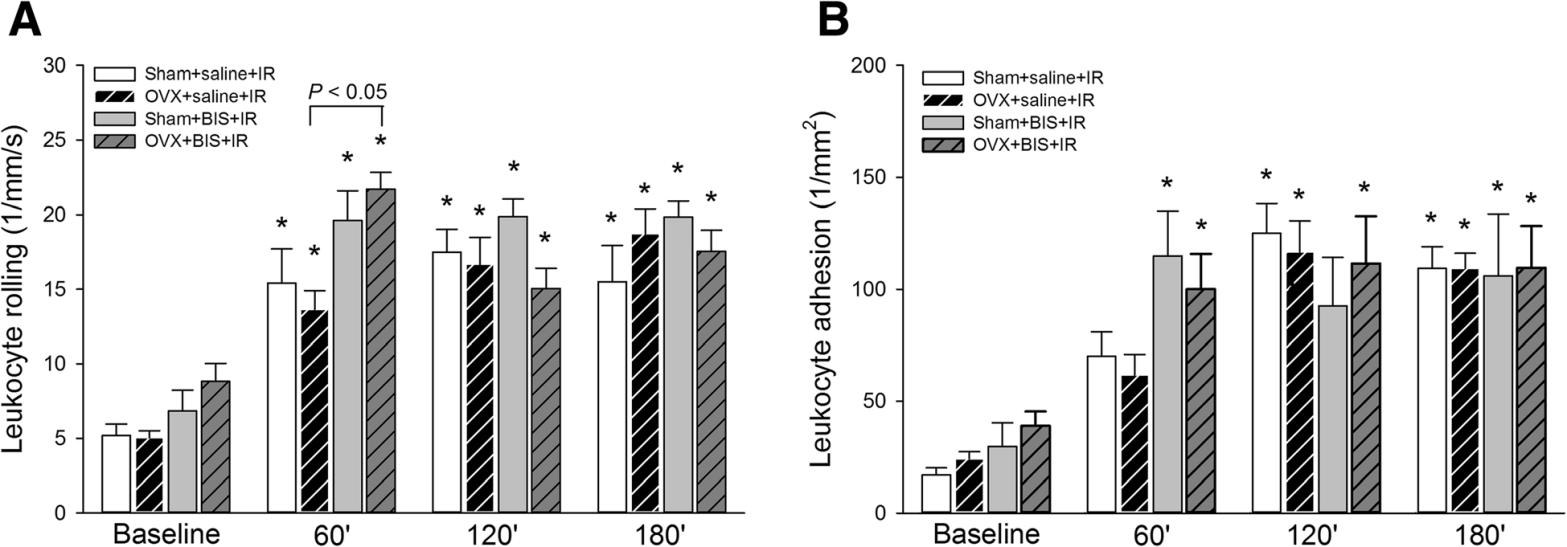
Changes in leukocyte rolling (**a**) and adhesion (**b**) in the tibial periosteal postcapillary venules in response to 60 min of tourniquet ischemia (IR) followed 60, 120 and 180 min of reperfusion in sham-operated (sham) and ovariectomized (OVX) rats treated with bisphosphonate (BIS) or a saline vehicle. Here, data values are given as means ± SEM, and *P < 0.05 vs baseline. Two-way RM ANOVA was followed by the Holm–Sidak and Dunn post hoc tests

Chronic ZOL treatment did not influence baseline values of leukocyte–endothelial interactions in the periosteal microcirculation (Fig. 2a, b). IR, however, induced significant increases in both PMN rolling and adhesion during the entire reperfusion period, and these changes reached a similar level in sham-operated and ovariectomized rats. BIS treatment caused a temporary increase in leukocyte rolling in OVX + IR animals and, similarly, an earlier rise in PMN adhesion in both sham + IR and OVX+IR animals at 60 min of reperfusion but did not influence PMN–endothelial interactions in later stages of reperfusion.

**Fig. 3:**
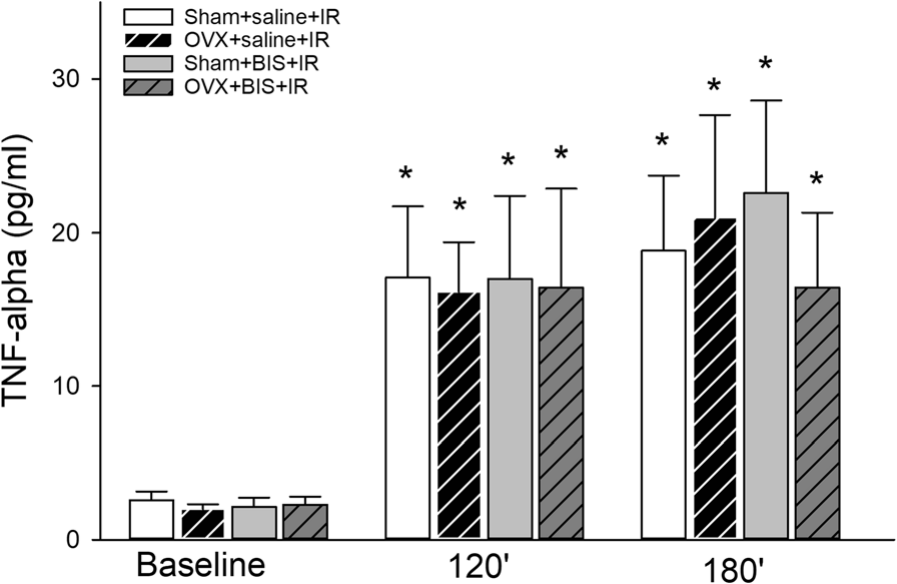
TNF-alpha levels in plasma samples at baseline and in response to 60 min of limb ischemia followed by 120 and 180 min of reperfusion (IR) in sham-operated (sham) and ovariectomized (OVX) rats treated with bisphosphonate (BIS) or a saline vehicle. Here, data values are given as means ± SEM, and *P < 0.05 vs baseline. Two-way RM ANOVA was followed by the Holm–Sidak post hoc test

As compared to baseline, TNF-alpha values showed marked increases during the reperfusion period under examination (Fig. 3). No differences could be traced among the different experimental groups.

**Fig. 4:**
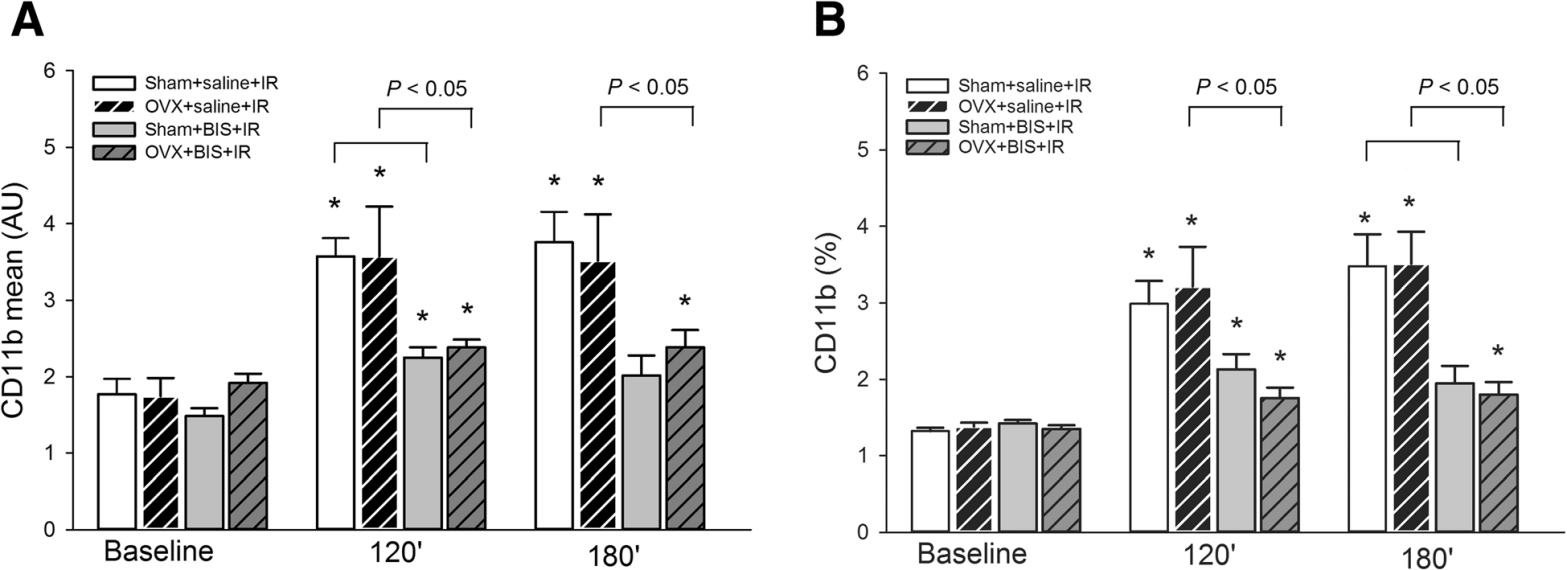
Changes in expression of the CD11b adhesion molecule on the surface of polymorphonuclear leukocytes (PMNs) expressed as mean fluorescence intensity values (**a**) and percentage of positive cells of the immune-labelled cells (**b**) within the gated PMN population. Values are shown at baseline and at 120 and 180 min of reperfusion after 60 min of limb ischemia in sham-operated (sham) and ovariectomized (OVX) rats treated with bisphosphonate (BIS) or a saline vehicle. Here, data values are given as means ± SEM, and *P < 0.05 vs baseline. Two-way RM ANOVA was followed by the Holm–Sidak and Dunn post hoc tests

In comparison to baseline values, the amount of adhesion molecule CD11b on the PMN surface significantly increased in saline-treated sham-operated and OVX rats during reperfusion (Fig. 4a, b). In animals that received chronic BIS treatment, however, this elevation reached a significantly lower level.

## Discussion

BISs are effective medications for bone metastases and osteoporosis and promising treatment modalities for complex regional pain syndrome upon fracture healing [19–21]. The use of ZOL has been shown to have a positive effect on spinal fusion [22] and to promote osseointegration and fixation of dental implants in autologous bone grafts in osteoporosis [23]. The periapical lesion-induced bone loss in the mandible was effectively ameliorated [24], and osseointegration of titanium implants in postmenopausal osteoporosis was promoted by ZOL [9]. Furthermore, ZOL brought about periosteal bone formation after tooth extraction in osteopenic sheep [25]. ZOL treatment, however, also induced reactive periosteal hypertrophy and even BIS-related osteonecrosis of the jaw in the same osteopenic sheep model [26]. Nevertheless, the effect of BIS on IR-induced local and systemic inflammatory reactions has not been examined elsewhere in an osteopenic model.

It is noteworthy that both anti- and proinflammatory effects have been attributed to different BIS compounds. The anti-inflammatory aspects of BISs include upregulation of the number of inflammatory monocytes [27], modulation of the proliferation and the viability and apoptosis of monocytes and macrophages [28, 29] and a downregulation of proinflammatory cytokines, such as TNF-alpha [30, 31], as well as other cytokines, such as IL-1, IL-6 and neurogenic growth factor [20]. Similarly, inhibitory effects of BIS against neurogenic inflammation have also been reported [20]. On the other hand, an acute phase response (< 3 days) was induced by different BISs including ZOL with increased TNF-alpha release in patients [32], but tissue accumulation of PMNs, increased TNF-alpha release and marked oxidative stress were also demonstrated in other tissues, such as the gingiva [33] and the liver [34] in animal models. Furthermore, priming of immunological reactions was also attributed to ZOL [35]. BISs cause ocular inflammatory complications in some clinical cases [36] and healing complications of the jawbones after invasive dental interventions even leading to osteonecrosis [10]. ZOL has been shown to aggravate kidney damage (by increasing cytokine production, metabolic acidosis and apoptosis) during IR injury in rats [37].

Enhanced leukocyte–endothelial interactions have been demonstrated after BIS treatment in an arthritis model in mice, but little is known on the ZOL-induced periosteal microcirculatory reactions [38]. Previously, we demonstrated that chronic BIS treatment induces some level of microcirculatory inflammation in the mandible, but such effects were not observed in the tibial periosteum [14]. Therefore, in this study, we tested the effect of chronic ZOL treatment in a tourniquet-induced limb ischemia model, where the role of PMN–endothelial interactions in the development of postischemic microcirculatory inflammatory reactions is well established. Here, we have shown that the reduced endogenous oestrogen levels evoked by OVX do not predispose to enhanced periosteal microcirculatory complications per se [39, 40], with the results also demonstrating that, apart from temporary exacerbation of PMN–endothelial interactions at the early stages of reperfusion, no major microcirculatory inflammatory risk could be detected after chronic ZOL treatment. Oestrogen withdrawal induces a release of TNF-alpha, which is involved in the pathomechanism of osteoporotic bone loss in women [41]; but here, we did not demonstrate between-group differences in TNF-alpha levels in the postischemic phase. Nevertheless, unlike humans, where increased serum TNF-alpha levels after OVX have been observed [42], we did not detect any differences between the baseline TNF-alpha levels between the different experimental groups. It should be noted that serum levels of TNF-alpha are rather low in rats and baseline values were close to the detection limit of the assay.

CD11b expression is a critical step for PMN adhesion to activated endothelial cells, and we detected a reduced IR-induced systemic PMN-derived CD11b expression after ZOL administration. BISs have been shown to influence PMN functions which manifested in impaired PMN chemotaxis and reactive oxygen species producing capacity in vivo [43] and reduced myeloperoxidase and NADPH oxidase activities in vitro [44, 45]. The inhibitory effect of BIS was also demonstrated in other immune cells such as macrophages [30]. In our study, ZOL reduced CD11b expression on the surface of circulating PMNs but did not influence the overall adhesion of PMNs in the periosteal postcapillary venules. This finding can only be explained by some degree of ZOL-induced endothelial activation and secondary endothelium-derived adhesion molecule expression. This possible ZOL-induced endothelial upregulation of adhesion molecules (the endothelial counterparts of CD11b) which might be responsible for the present results should be further investigated.

Among other effects, BISs are known to inhibit vascular endothelial proliferation and to upregulate cellular apoptosis [46]. Furthermore, BISs (alendronate) have also been shown to inhibit nitric oxide synthase expression, which is an important endogenous modulator of PMN–endothelial interactions [47]. These ZOL-induced acute postischemic reactions affecting the endothelium may also warrant further in-depth investigations.

In summary, BIS treatment exerted only a minor influence on limb IR-induced PMN rolling and adhesion in the periosteum, and the PMN-derived adhesion molecule (CD11b) expression on circulating PMNs was even reduced. Further, no effect on postischemic TNF-alpha release was demonstrated in ZOL-treated rats. These results suggest that although some level of local endothelial activation might be attributable to the treatment, chronic ZOL administration does not have a major influence on the risk of postischemic inflammatory microcirculatory complications in the tibial periosteum.

## Abbreviations

BIS: Bisphosphonate
IR: Ischemia–reperfusion
IVM: Fluorescence intravital microscopy
OVX: Ovariectomy
PMN: Polymorphonuclear leukocytes
ZOL: Zoledronic acid
